# Influence of the Season and Region Factor on Phosphoproteome of Stallion Epididymal Sperm

**DOI:** 10.3390/ani11123487

**Published:** 2021-12-07

**Authors:** Katarzyna Dyrda, Aleksandra Orzołek, Joanna Ner-Kluza, Paweł Wysocki

**Affiliations:** 1 Department of Animal Biochemistry and Biotechnology, Faculty of Animal Bioengineering, University of Warmia and Mazury in Olsztyn, M. Oczapowskiego 5, 10-719 Olsztyn, Poland; k.mietelska@wp.pl (K.D.); aleksandra.deszczka@uwm.edu.pl (A.O.); 2 Department of Biochemistry and Neurobiology, Faculty of Materials Science and Ceramics, University of Science and Technology, A. Mickiewicza 30, 30-059 Krakow, Poland; nerkluza@agh.edu.pl

**Keywords:** stallion, epididymis, phosphoproteins, reproductive season, sperm maturation

## Abstract

**Simple Summary:**

Phosphorylation and dephosphorylation of proteins are considered to be the most important processes in sperm maturation during the epididymal transit. We demonstrated that 27 proteins underwent phosphorylation both in and out of the breeding season. Differences in the phosphorylation status were demonstrated in the case of endoplasmic reticulum chaperone BiP, albumin, protein disulfide-isomerase A3, nesprin-1, peroxiredoxin-5, and protein bicaudal D homolog.

**Abstract:**

Epididymal maturation can be defined as a scope of changes occurring during epididymal transit that prepare spermatozoa to undergo capacitation. One of the most common post-translational modifications involved in the sperm maturation process and their ability to fertilise an oocyte is the phosphorylation of sperm proteins. The aim of this study was to compare tyrosine, serine, and threonine phosphorylation patterns of sperm proteins isolated from three subsequent segments of the stallion epididymis, during and out of the breeding season. Intensities of phosphorylation signals and phosphoproteins profiles varied in consecutive regions of the epididymis. However, significant differences in the phosphorylation status were demonstrated in case of endoplasmic reticulum chaperone BiP (75 and 32 kDa), protein disulfide-isomerase A3 (50 kDa), nesprin-1 (23 kDa), peroxiredoxin-5 (17 kDa), and protein bicaudal D homolog (15 kDa) for season x type of phosphorylated residues variables. Significant differences in the phosphorylation status were also demonstrated in case of endoplasmic reticulum chaperone BiP and albumin (61 kDa), protein disulfide-isomerase A3 (50 kDa), and protein bicaudal D homolog (15 kDa) for region x type of phosphorylated residues variables.

## 1. Introduction

Mammalian spermatozoa leave the testes as immobile and functionally immature reproductive cells. Gametes obtain motility and fertilisation potential during their transit through the epididymal duct. While there, spermatozoa undergo a series of poorly characterised post-translational modifications [1]. All alterations that occur during the maturation of male gametes are aimed at preparing cells for capacitation, acrosome reaction, and remodelling of the sperm surface so as to enable the sperm–oocyte fusion. Such changes include redistribution or disappearance of some polypeptides, as well as the action of glycolytic enzymes and integration of newly synthesised components [2].

It is well known that maturation of epididymal spermatozoa is associated with the activation of a cAMP-induced tyrosine phosphorylation cascade. During the epididymal transit, the level of sperm’s intracellular cAMP gradually increases from the corpus to the cauda, and so does the metabolic capacity and ATP production [3]. These changes are associated with the subsequent phenomena as hyperactivation of the motility and acrosomal exocytosis of the sperm. As spermatozoa move through the epididymis, tyrosine targets localised on the principal piece of spermatozoa are phosphorylated first. Next, phosphorylation embraces the midpiece of sperm. By the time gametes will have reached the cauda of the epididymis, they are subjected to phosphorylation within the entire tail, from the neck to the endpiece. This particular pattern of phosphorylation is associated with the sperm maturation, a requirement for acquiring competence for fertilisation [1]. For instance, evaluation of the tyrosine phosphorylation pattern is important for understanding the further process of sperm maturation that includes acquisition of mobility, changes in sperm plasmalemma, mitochondrial activity, resistance to oxidative stress, and finally fertilisation of the oocyte [4]. Most importantly, phosphorylation of tyrosine residues of the sperm tail induces the hyperactivation of sperm [5]. The differentiated abundance of sperm phosphoproteins was demonstrated in two human sperm populations with high and/or low mobility [6]. What is interesting is that PSer/PThr residues also take part in regulation of sperm motility [7]. The phosphorylation of proteins on serine or threonine residues plays an essential role in the regulation of cellular processes such as cell proliferation and differentiation [8]. Furthermore, phosphorylation of serine and/or threonine residues is known to bring the conformational changes to the protein and regulate it by bringing them. Phosphorylated serine/threonine residues can also function as binding motifs for recruiting proteins into signalling networks or placing enzymes within proximity to substrates [9]. Phosphorylation/dephosphorylation processes can influence the structure of proteins at both local and global levels. Phosphorylation may trigger the transition between conformations and lead to the activation or deactivation of a chosen protein [10].

Abiotic factors such as temperature and photoperiodism are important factors that might influence both the morphology and the function of spermatozoa [11]. In the stallion, photoperiod-driven serum concentrations of FSH, LH, testosterone, and prolactin are the highest in the summer, the same as the testicular weight, intratesticular testosterone levels, number of Sertoli and Leydig cells, number of spermatogonia, and daily sperm production [12]. In seasonally reproducing animals, the morphology and function of the epididymis change similarly to the testicular tissue. On the one hand, such modification is an adaptation to the environment, and on the other, it minimises the energetic effort needed for reproduction [13].

The aim of this study was to compare tyrosine, serine, and threonine phosphorylation patterns of sperm proteins isolated from three subsequent tracts of the stallion epididymis, during and out of the breeding season.

## 2. Materials and Methods

All chemicals were purchased from Sigma-Aldrich (St. Louis, MO, USA), unless otherwise stated.

### 2.1. Material Collection

The research material consisted of 12 epididymides, which were obtained from sexually mature (3–4 years), warmblood stallions during castration surgeries. All individuals were born in the same horse ranch, and they stayed there until the moment of castration occurred. The horse farm is situated in Zastawno village and possesses the following geographical coordinates: the longitude 54.178358437261245° N and the latitude 19.632087055009112° E. Surgical procedures were performed in the breeding season, i.e., from March to June (*n* = 6), and out of the breeding season, i.e., from September to December (*n* = 6). Promptly after the surgeries, epididymides were thoroughly rinsed with 0.85% NaCl, placed in sterile packs, and transported in a thermobox (5 °C) to the laboratory.

**Figure 1:**
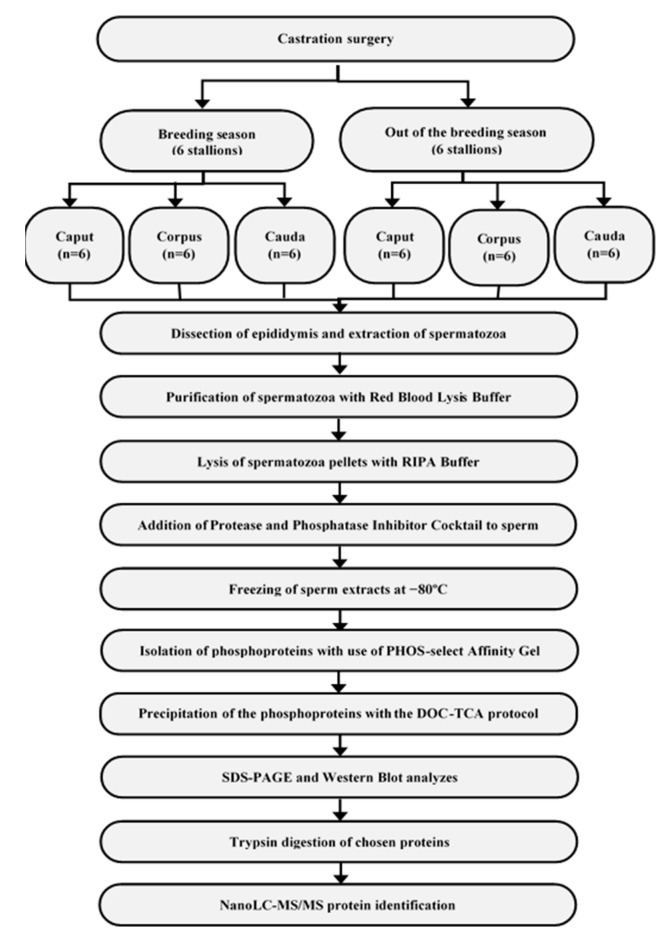
The scheme presenting the step-by-step course of action taken during the survey conducting.

All methodological approach taken during the experiment is presented above (Figure 1). First, epididymides were dissected into three anatomically separate parts, i.e., the caput, cauda, and corpus. The individual segments of each epididymis were cut into pieces (0.25 cm^3^), then suspended in 0.85% NaCl and centrifuged twice at 2000× *g* for 10 min at 10 °C [14]. The supernatant with epididymal spermatozoa was transferred into another test tube and centrifuged at 10,000× *g* for 5 min at 10 °C. In order to avoid contamination by erythrocytes, Red Blood Cell Lysing Buffer was added to every sample. Then, the precipitations were diluted in RIPA buffer (50 mM Tris-HCl, 150 mM NaCl, 1% Triton X-100, 0.5% sodium deoxycholate, 0.1% SDS; pH 8.0). Moreover, Protease and Phosphatase Inhibitor Cocktail was added to every test tube. Samples were frozen at −80 °C. After thawing, sperm extracts were centrifuged at 10,000× *g* for 10 min at 24 °C. The total protein content was determined in every sample [15].

### 2.2. Isolation and Precipitation of Phosphoproteins

Phosphoproteins were isolated on PHOS-select Iron Affinity Gel. The isolation procedure was performed with equilibrating solution (250 mM acetic acid with 30% acetonitrile) and elution solution (400 mM ammonium hydroxide). Samples containing 0.5 mg of total protein were applied onto the columns and subjected to phosphoprotein isolation. Fractions of 100 μL in volume were eluted from the columns to new test tubes.

Phosphoproteins were precipitated according to the DOC-TCA protocol [16], with modifications. Briefly, 1 μL of 2% sodium deoxycholate was added to 100 μL of every sample and incubated for 30 min at 4 °C. Next, all samples were centrifuged (15,000× *g*, 15 min, 4 °C) and dried in speed vacuum. The pellets were suspended in SDS-loading buffer (2% SDS, 5% β-mercaptoethanol, 125 mM Tris-HCl; pH 6.8).

### 2.3. SDS-PAGE and Western Blotting

Phosphoproteins were separated by SDS-PAGE according to the method by Laemmli [17] and stained by Coomassie Brilliant Blue R-250. A total of 20 μL of every sample was applied onto a single gel path. Precision Plus Protein Standard (Bio-Rad, Hercules, CA, USA) served as a molecular weight reference. Molecular weights of phosphoprotein fractions were estimated with the use of Multi-Analyst software (Bio-Rad, USA).

Phosphoproteins were electro-transferred to PVDF membranes (Millipore, Burlington, MA, USA) using Semi Dry Blotter (Sigma-Aldrich, St. Louis, MO, USA). Therefore, membranes were blocked in TBS (10 mM Tris-HCl, 100 mM NaCl, 0.1% Tween 20; pH 7.5), with 2% bovine serum albumin, for 1 h at 4 °C. After blocking, the membranes were washed four times for 5 min in TBS buffer and left for overnight incubation at 4 °C in TBS with the addition of monoclonal biotinylated antibodies, either anti-phosphotyrosine, anti-phosphoserine, or anti-phosphotreonine antibodies (Sigma-Aldrich, USA) in 1:50,000, 1:30,0000, or 1:60,000 dilutions, respectively. On the subsequent day, the membranes were washed with TBS buffer three times for 5 min and incubated for 2 h in TBS buffer (20 mL) with the addition of streptavidin-alkaline phosphatase (4 μL). Afterwards, they were washed four times in TBS buffer for 5 min and stained in 10 mL of buffer containing 100 mM Tris-HCl and 100 mM NaCl (pH 9.5) with the addition of 200 μL of NBT-BCIP solution until stained bands were visible. Molecular masses of fractions were assessed using Multi-Analyst software (Bio-Rad, USA).

### 2.4. Trypsin Digestion of Chosen Proteins

Selected proteins that were characterised by different intensities of phosphorylation in subsequent segments of the epididymis were subjected to in-gel trypsin digestion after SDS-PAGE. Excised gel pieces were washed with 50% acetonitrile (ACN)/0.1% trifluoroacetic acid (TFA) once. Then, they were destained twice with 200 µL of ammonium bicarbonate (100 mM) and dehydrated with 100 µL of acetonitrile (50%) at 37 °C. Ultimately, they were dried in speed vacuum. Thereafter, they were subjected to reduction and alkylation process using first 50 mM dithiothreitol and next 50 mM iodoacetamide/50 mM NH_4_HCO_3_ solutions. Then, the samples were incubated with 100 μL of 50 mM NH_4_HCO_3_ at room temperature for 5 min with gentle shaking. Afterwards, they were centrifuged, dehydrated with 100 µL of 50% can, and dried in speed vacuum. Thereafter, samples were incubated overnight at 37 °C with trypsin (12.5 ng/µL in 25 mM NH_4_ HCO_3_) (Promega, Madison, WI, USA). Peptides were extracted twice with 25 µL of formic acid in 50% ACN for 30 min at 37 °C with sonification. The extracts were dried in speed vacuum again and resuspended in 20 µL of 5% ACN with 0.1% addition of formic acid. Tryptic digests were analysed by the NanoLC–MS/MS technique.

### 2.5. NanoLC–MS/MS Protein Identification

A nanoLC–MS/MS analysis was made on a Proxeon EASY-nLC II nanoLC system (Thermo Fisher Scientific, Dreieich, Germany) connected online to an ESI-IT mass spectrometer (AmaZon ETD, Bruker-Daltonics, Bremen, Germany) operated in a positive-ion mode. A total of 7 µL of a sample was loaded on a two-column system: an RP C18 precolumn (2 cm, 5 μm particle size, 100 μm ID, Thermo Fisher Scientific, Waltham, MA, USA) and an RP C18 separation column (10 cm, 3 μm particle size, 75 μm ID, Thermo Fisher Scientific, USA). The flow rate was set at 300 nL/min. Peptides were eluted at 70 min long gradient of buffer A (0.1% formic acid in water) and B (0.1% formic acid in acetonitrile). Gradient settings were as follows: time t = 0 min 98% A, t = 50 min 50% A, t = 50,1 min 10% A, t = 55 min 10% A, t = 55,1 min 10% A, t = 70 min 2% A. Data Analysis software (version 4.0, Bruker-Daltonics, Germany) was used to convert raw data to mgf files suitable for protein identification by Mascot software. All mgf files were searched against the mammalian taxonomic group in the UniProt KB database. Other settings included the enzyme used, i.e., trypsin (1 missing cleavage acceptable); fixed modifications: carbamidomethylation; variable modifications: met-oxidation, phosphorylation (S, T, Y), peptide tolerance ±1.2 Da; MS/MS tolerance: ±0.8 Da; peptide charge: 1+, 2+, 3+; instrument: ESI-TRAP. Search was performed by Mascot software, (Matrix Science, London, United Kingdom). Scores ≥ 50 were regarded as significant according to Mascot software. The results were given in the summary table (Table 1).

### 2.6. Statistical Analysis

Statistical analyses were conducted with the use of Statistica programme (version 13.1, StatSoft Incorporation, USA). Three variables were taken into consideration in all analyses, i.e., season, segment of the epididymis, and type of antibodies. The data were analysed by ANOVA, followed by non-parametric Mann–Whitney *U* (for season × antibody variables) and Kruskal–Wallis (for segment × antibody variables) tests. The results were shown as medians and standard deviations (SD) (Table 2 and Table 3).

## 3. Results

**Figure 2:**
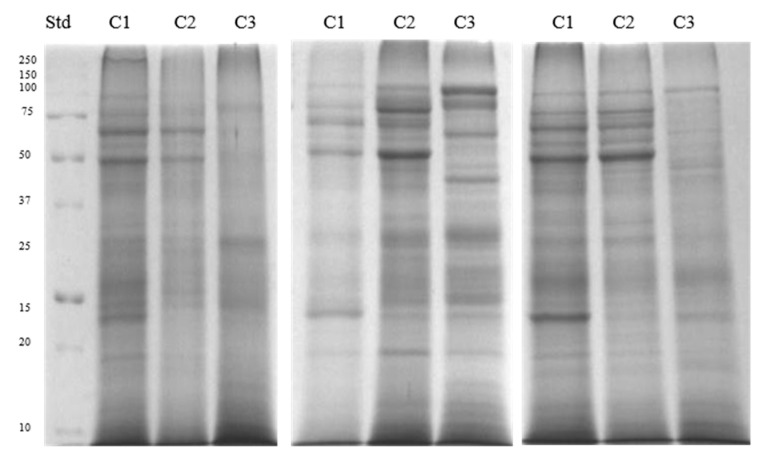
SDS-PAGE profile of sperm extracts proteins obtained during the breeding season. C1—caput, C2—corpus, C3—cauda. Std.—molecular weight standards.

**Figure 3:**
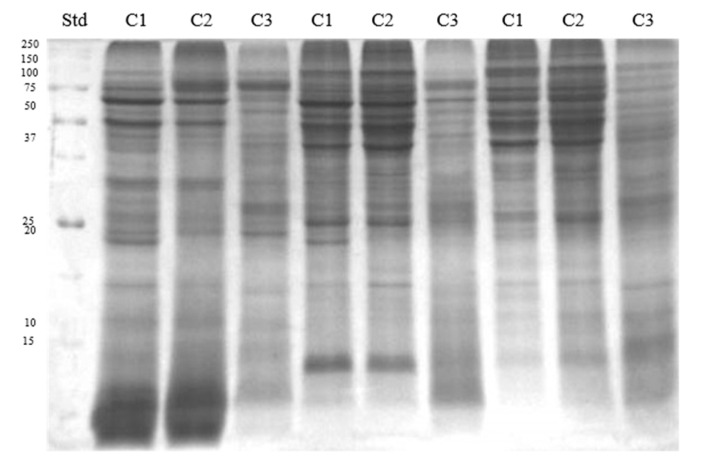
SDS-PAGE profile of sperm proteins obtained during out of the breeding season. C1—caput, C2—corpus, C3—cauda. Std.—molecular weight standards.

**Figure 4:**
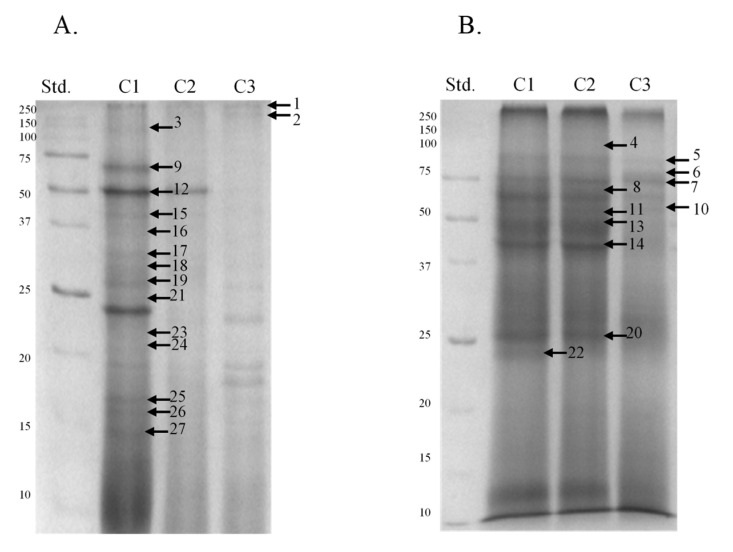
SDS-PAGE profile of phosphoproteins obtained during the breeding season (**A**) and out of the breeding season (**B**). A total of 27 arrows present proteins identified by nanoLC–MS/MS spectrometry. C1—caput, C2—corpus, C3—cauda. Std.—molecular weight standards.

**Figure 5:**
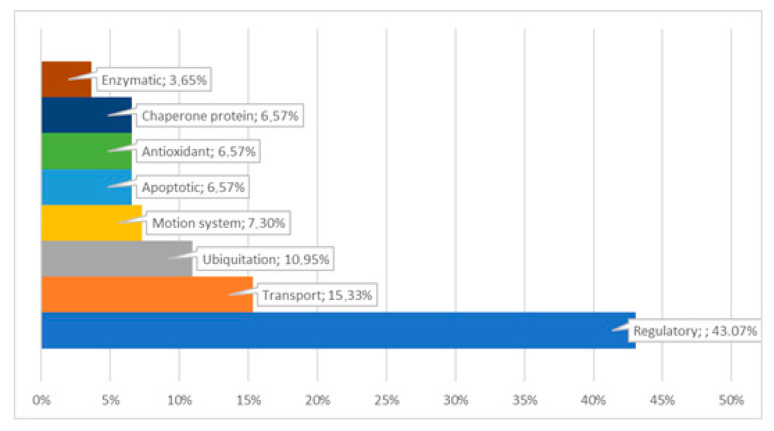
Biological functions of identified phosphoproteins derived from stallion epididymal sperm.

Electrophoretic profiles of separated sperm proteins and phosphoproteins showed the highest number of fractions in the caput and corpus segments, both in (Figure 2 and Figure 4A) and out of the breeding season (Figure 3 and Figure 4B). However, a greater quantity of protein fractions was observed during the breeding season. In the SDS-PAGE profiles of full sperm extracts, obtained in season, we observed approximately 24, 22, and 19 protein fractions in caput, corpus, and cauda regions, respectively (Figure 2), whereas full sperm extracts, gained in the time called off-season, were characterised by the presence of approximately 21 in caput, 19 in corpus, and 18 protein fractions in cauda region (Figure 3). It should be highlighted that protein profiles of C1 and C2 regions differed more significantly during the time of the season (Figure 2). On the other hand, SDS-PAGE profiles of sperm derived from C1 and C2 regions, gained out of the season, were more similar to each other (Figure 3). We observed 27 proteins fractions whose presence was differentiated among segments of the epididymis and individuals (Figure 4). All aforementioned proteins were subjected to mass spectrometry (Table 1). Polypeptides that were predicted by MS were grouped together in compliance with the biological role they serve (Figure 5).

**Figure 6:**
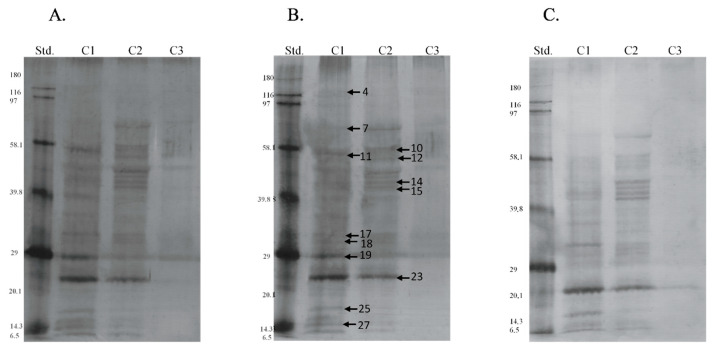
Western blot analysis of phosphoserine (**A**), phosphothreonine (**B**), and phosphotyrosine (**C**) residues obtained from sperm extracts during the breeding season. Std.—Biotinylated Molecular Weight Protein Standards (Sigma-Aldrich). C1—caput, C2—corpus, C3—cauda. Phosphoproteins identified by mass spectrometry are marked with arrows.

**Figure 7:**
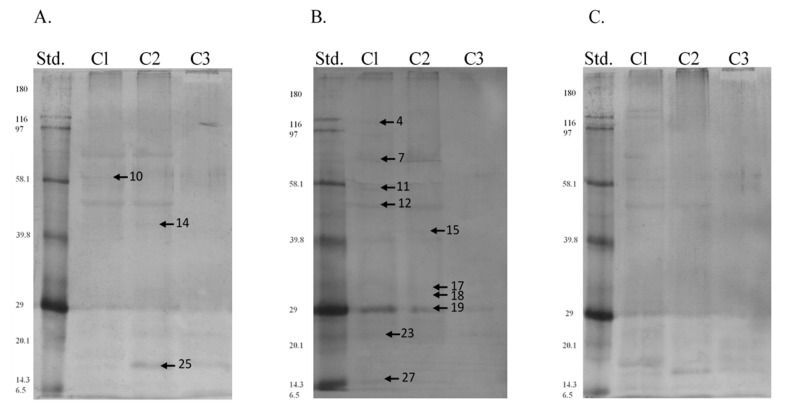
Western blot analysis of phosphoserine (**A**), phosphothreonine (**B**), and phosphotyrosine (**C**) residues obtained from sperm extracts out of the breeding season. Std.—molecular weight standards. C1—caput, C2—corpus, C3—cauda. Phosphoproteins identified by mass spectrometry are marked with arrows (see Table 1).

Interestingly, the protein profiles that were obtained after Western blots and immunoblottings did not coincide completely with the results of electrophoreses. This might have been caused by various degrees of residue phosphorylation that yielded varied signal intensities. We demonstrated that all the antibodies applied detected almost the same phosphorylation patterns in the stallion sperm. However, we also noticed some season- and segment-dependent patterns of phosphorylation of some proteins. We demonstrated that 13 out of 27 phosphoprotein fractions previously identified, i.e., 110, 75, 61, 57, 50, 44, 41, 32, 31, 29, 23, 17, and 15 kDa, yielded the strongest signal intensification (Figure 6 and Figure 7). We confirmed significant differences in the degree of phosphorylation in case of 75, 50, 32, 23, 17, and 15 kDa polypeptides for season x type of phosphorylated residue variables (Table 2), and 61, 50, and 15 kDa polypeptides for region x type of phosphorylated residue variables (Table 3). Profiles of the remaining phosphoproteins were differentiated among individuals and seasons.

## 4. Discussion

Post-translational modifications through serine/threonine or tyrosine phosphorylation by protein kinases, and/or the dephosphorylation of mentioned residues by phosphoprotein phosphatases, play a major role in the transduction of extracellular signals, intracellular transport, and cell cycle progression [18]. Phosphorylation at specific residues can activate a protein, lead to its localisation in particular compartments, or induce protein degradation [19]. Phosphorylation is not the only regulatory system in the cell. Conversely, it is associated with many other systems, e.g., ubiquitin ligases, GEFs, actin-binding proteins, and RNA-interacting proteins. The main advantage of following the dynamics of phosphosites alterations rather than whole proteins is that signalling outcomes can be more directly connected to responsible upstream or downstream events [20]. The phosphorylation of a receptor molecule not only activates various signalling cascades but may also deactivate the processes in further stages [21]. Generally, phosphorylated serine residues are the most abundant (86%), followed by threonine (12%) and tyrosine phosphorylations (2%) [22].

In our study, we demonstrated that the majority of the epididymal sperm proteins undergo phosphorylation on serine, threonine, and/or tyrosine residues, regardless of the time of the year. Phosphorylation of residues of endoplasmic reticulum chaperone BiP, protein disulfide-isomerase A3, nesprin-1, peroxiredoxin-5, and protein bicaudal D homolog 2 was enhanced by the season. On the other hand, phosphorylation of residues of endoplasmic reticulum chaperone BiP, albumin, protein disulfide-isomerase A3, and protein bicaudal D homolog 2 was influenced by the region factor. Moreover, we showed that the phosphoproteome of stallion epididymal spermatozoa is composed of various groups of proteins, including elongation factors, chaperones, hydrolases, transporters, enzyme modulators, and cytoskeletal proteins, suggesting their essential role in the stallion sperm maturation. Phosphoproteins derived from the stallion epididymal sperm were previously found in epididymides of different species, e.g., human, bovine, and mouse [23,24,25].

Five polypeptides identified in our research were components of numerous protein bands obtained after 1D SDS-PAGE and subjected to mass spectrometry. Titin (TTN) was predicted in 21, endoplasmic reticulum chaperone BiP in 14, dystonin in 6, inactive ubiquitin carboxyl-terminal hydrolase-54 in 5, and nesprin-1 in 4 out of 28 phosphorylated bands present in the stallion epididymal sperm extracts.

The exact function of titin (TTN) in tissues other than muscle has not been established until now. Certainly, TTN can potentially associate with more than 20 different polypeptides [26]. These authors suggested that titin could be an important regulatory node and a component of the mechanism that balances protein folding and degradation. Phosphorylation sites on titin may be involved in regulating the aforementioned pathways. TTN with the molecular weight of 3827.5 kDa and pI 6.35 was identified as a constituent of the seminal plasma proteome in fertile men [27].

BiP is a member of HSP70 chaperone family located in the ER. It can interact with both non-glycosylated and glycosylated proteins. BiP is responsible for maintaining the permeability barrier of the ER during protein translocation as well as targeting misfolded proteins for retrograde translocation [28]. Dystonin (DST) is a large cytolinker protein of the plakin family that plays a crucial role in the cytoskeletal organisation, organelle integrity, and intracellular transport [29]. It was previously found in seminal plasma of fertile men [27], although DST, like isoform 1, underwent over-expression among low-fertility bulls [30].

In contrast, little is known about the expression and physiological roles of deubiquitinating enzymes in male germ cells, and only few have been identified as important regulators of spermatogenesis thus far [31]. Ubiquitin carboxyl-terminal hydrolase is incapable of processing large ubiquitinated products. This enzyme cleaves ubiquitin from small adducts, and its expression is associated with the maintenance of free ubiquitin pool inside tissues [32]. The ubiquitous presence of TTN, BiP, and ubiquitin carboxyl-terminal hydrolase in stallion epididymal sperm may be associated with their contribution to the system of detecting and removing incorrectly synthesised proteins as well as their binding properties. Presence of DST may be attributed to both its regulatory potential and participation in cytoskeleton forming. On the other hand, occurrence of nesprin-1 in many samples may be explained by its participation in sperm nucleus forming [33].

During the breeding season, more intensive phosphorylation degrees were observed in the case of bands with 75, 50, 32, 23, 17, and 15 kDa. The protein predicted as endoplasmic reticulum chaperone BiP (75 kDa, band 7) was subjected to stronger phosphorylation (*p* ≥ 0.05) on threonine residues during this time. The protein disulphide isomerase family A3 (PDIA3) (50 kDa, band 12) has an activity of thioredoxins, and is widely distributed among multiple tissues. PDIA3 has recently been found in human and mouse sperm cells. It may affect the sperm–egg fusion [34]. Although PDIA3 was initially described as a resident protein of the endoplasmic reticulum (ER), it was also shown on the cell surface [35]. Ellerman et al. [36] speculated that the disulphide isomerase activity of PDIA3 may interact with the disulphide bonds of Izumo1, CD9, or CRISPs to cause conformational change in these proteins and thereby induce the sperm membrane to fuse with the egg membrane. We demonstrated that stronger phosphorylation of PDIA3 embraced mainly serine residues.

Peroxiredoxins (PRDXs) (17 kDa, band 25) regulate ROS levels in almost all cell types. They maintain cell homeostasis by regulating mainly H_2_O_2_ levels [37]. Decreased PRDX activity in sperm is associated with a significant reduction of motility parameters, viability, and intracellular ATP content [38]. A 17.5 kDa polypeptide is secreted from the cauda epididymis, and it binds to the cauda sperm plasma membrane during epididymal transit. Proteomic identification of the 17.5 kDa polypeptide yielded 13 peptides that matched the sequence of peroxiredoxin-5 (PRDX5) protein (Bos taurus). It was proposed that bovine cauda sperm PRDX5 acts as an antioxidant enzyme in the cauda epididymal environment to protect the viable sperm population against the damage caused by endogeneous or exogeneous peroxide [39]. Robust phosphorylation of peroxiredoxin-5 in stallion sperm within the breeding season may be associated with its protective antioxidant activity.

Protein bicaudal D homolog 2 (BICD2) (15 kDa, band 27) is linked to the trans-Golgi network, which in turn binds to the dynamitin subunit of dynactin [40]. Dynactin acts as an adaptor between motor proteins and cargo to facilitate the transport of membrane vesicles. Missense mutations in BICD2 have been identified in patients with congenital autosomal-dominant spinal muscular atrophy (SMA) [41]. We observed that during the breeding season, it was subjected to stronger phosphorylation on threonine residues and that its phosphorylation took place mainly in the caput and corpus regions.

Other proteins that underwent intensified phosphorylation both in and off the breeding season included elongation factor 1-alpha, microtubule-actin cross-linking factor 1, heat shock proteins, and endoplasmin. There were no significant differences in their phosphorylation degrees within both periods, although their presence was differentiated between the animals. This might be associated with various activities of epididymides of individuals. Phosphorylation of chosen sperm proteins appears to be related also to the specific region of stallion epididymis. We noticed that more intensive phosphorylation involved protein bands of 61, 50 (PDIA3, band 12), and 15 kDa (BICD2, band 27).

Among 61 kDa band centromere-associated protein E (CENP-E) and DNA-dependent protein, kinase catalytic subunits were predicted. Centromeric protein E, a kinesin-7 family member, plays a key role in the movement of chromosomes during mitosis. It plays an essential role in capturing and positioning chromosomes during metaphase. CENP-E is localised on chromosomes and remains there until anaphase, at which point it is relocated and subsequently degraded [42]. We ascertained that 61 kDa underwent statistically stronger phosphorylation on tyrosine residues in the cauda region. The band with 50 kDa within which PDIA3 was predicted underwent phosphorylation on threonine residues, whereas 15 kDa (BICD2) was phosphorylated on serine and tyrosine residues, mainly in the caput and corpus regions.

The patterns of sperm phosphorylation may be associated with the functional states in which proteins occur. Sometimes, polypeptides possess dynamic conformational ensembles that may contain noticeable regions of unstable tertiary and/or secondary structure. Such regions can be easily modified by phosphorylation [43]. On the one hand, disorder facilitates the access of kinase to the residue. On the other hand, the addition of a phosphate moiety may lead to a structural change. Disorder is strongly associated with protein–protein interactions. Modified residues found within disordered regions can act as on/off switches, either promoting or inhibiting interaction. Disorder and interfacial location of a polypeptide are significantly associated with phosphorylation of serine and, to a lesser extent, with phosphorylation of threonine. Conversely, tyrosine phosphorylation is often observed in ordered interface regions. The fractions of phosphorylated Ser, Thr, and Tyr in disordered interfaces are 59, 26, and 15%, respectively. Clustered pSer/pThr sites are usually phosphorylated by the same kinase, and clustered Ser/Thr prefer to be located in disordered regions [21].

Phosphorylation processes may also be dependent on the season. Certain enzymes, such as acid phosphatase and alkaline phosphatase, are highly sensitive to seasonal changes, and their activity coincides with the production of proteins in epididymis. The number of protein fractions present in epididymal lumen is the highest during the breeding season [44].

It seems that specific sperm surface proteins are removed or further processed as the gametes pass through the epididymis. The disappearance of some proteins is probably linked to a proteolysis that occurs during epididymal transit. Proteolysis induces either a change in the protein membrane domain distribution or release the protein into the epididymal fluid [45]. Higher number of surface proteins derived from caput spermatozoa may be associated with the increase in the rigidity of sperm membrane during maturation, which may be required for fertility of spermatozoa [46]. However, densitometric analysis indicated that the total amount of, for instance, sulphydrylated proteins in samples obtained from the stallion caput, corpus, and cauda epididymal regions was similar [47].

In conclusion, stallion sperm phosphoprotein patterns seem to be influenced by the season (endoplasmic reticulum chaperone BiP, protein disulfide-isomerase A3, nesprin-1, peroxiredoxin-5, protein bicaudal D homolog 2) and the region of the epididymis (endoplasmic reticulum chaperone BiP, albumin, protein disulfide-isomerase A3, protein bicaudal D homolog 2). The most probable explanation of such phenomenon is that the activity and physiology of the stallion epididymis changes according to the time of the year. Not only it is an adaptation to environmental conditions, but also a reduction of an energetic effort necessary for maintenance of reproductive functions. Basic data clarifying the seasonal variations in sperm phosphoproteome composition are essential for knowing the reproductive physiology of the stallion better.

## Figures and Tables

**Table 1:** Phosphoproteins identified by nanoLC–MS/MS spectrometry.

Band	Identified Protein	M. W. (kDa)		pI	Score	Sequence Cov. %	Peptide Matches
		MultiAnalyst	Mascot				
1	Elongation factor 1-alpha	280	50.1	9.7	83.9	4.1	2
	Titin		3904.1	5.8	63.2	0.2	6
	Phosphate carrier protein, mitochondrial		39.4	10.1	57.0	3.4	1
2	Titin	160	3813.7	6.0	81.3	0.2	7
3	Titin	150	3813.7	6.0	72.4	0.3	8
	Endoplasmic reticulum chaperone BiP		72.3	4.9	59.9	3.8	2
4	Endoplasmin	110	92.4	4.6	89.6	4.6	4
	Titin		3813.7	6.0	81.4	0.4	10
	Leucine-rich repeat ser/thr-protein kinase 2		285.9	6.4	59.3	1.5	2
5	Titin	100	3904.1	5.8	57.4	0.2	6
	Spectrin beta chain, non-erythrocytic 1		274.1	5.3	56.1	0.8	2
6	Endoplasmic reticulum chaperone BiP	83	72.3	4.9	107.1	3.2	4
	Titin		3813.7	6.0	81.4	0.4	7
7	Endoplasmic reticulum chaperone BiP	75	72.3	4.9	63.5	22.5	2
8	Endoplasmic reticulum chaperone BiP	73	72.3	4.9	174.0	11.0	6
	Serum albumin		68.5	5.9	77.2	3.6	2
9	Heat shock-related 70 kDa protein 2	70	69.6	5.4	141.3	9.8	6
	Heat shock 70 kDa protein 1-like		70.3	6.0	137.8	1.9	1
	Titin		3813.7	6.0	107.5	0.2	8
	Serum albumin		68.5	5.9	98.6	6.9	4
	Endoplasmic reticulum chaperone BiP		72.3	4.9	54.9	4.1	2
	Fibrous sheath-interacting protein 2		780.1	6.3	52.0	0.5	3
10	Serum albumin	61	68.6	5.9	362.0	13.8	10
	Endoplasmic reticulum chaperone BiP		72.3	4.9	156.8	9.0	4
	Titin		3813.7	6.0	69.7	0.3	7
	Centromere-associated protein E		286.3	5.1	54.7	1.4	4
11	Protein disulfide-isomerase A3 (Fragments)	57	23.4	4.7	54.5	11.1	2
12	Endoplasmic reticulum chaperone BiP	50	72.3	4.9	162.6	7.8	5
	Protein disulfide-isomerase A3		56.9	6.2	136.3	10.9	5
	Titin		3813.7	6.0	82.5	0.3	8
	Protein disulfide-isomerase		56.9	4.7	73.7	8.1	4
	BCL-6 corepressor-like protein 1		190.4	9.0	64.0	3.4	5
13	Serum albumin	48	68.5	5.9	69.4	4.0	2
	Microtubule-actin cross-linking factor 1, isoforms 1/2/3/5		837.8	5.3	57.1	0.6	4
14	Endoplasmic reticulum chaperone BiP	44	72.3	4.9	163.1	12.8	7
	Titin		3813.7	6.0	85.5	0.2	8
	Protein disulfide-isomerase		56.9	4.7	60.9	3.3	2
15	Endoplasmic reticulum chaperone BiP	41	72.3	4.9	234.6	11.3	5
	Titin		3813.7	6.0	81.7	0.2	7
	Protein disulfide-isomerase A6		48.1	4.9	80.5	3.4	1
	Protein TALPID3		169.2	5.4	51.0	1.8	2
	E3 ubiquitin-protein ligase HECTD1		289.2	5.2	50.8	1.8	4
16	Endoplasmic reticulum chaperone BiP	35	72.3	4.9	109.6	5.0	3
	Protein disulfide-isomerase A6		48.1	4.9	60.5	3.4	1
	Titin		3813.7	6.0	54.7	0.2	5
17	Endoplasmic reticulum chaperone BiP	32	28.9	4.6	77.4	7.3	2
18	Endoplasmic reticulum chaperone BiP	31	72.3	4.9	158.0	8.6	4
	Dystonin		860.1	5.1	75.3	0.5	3
19	Endoplasmic reticulum chaperone BiP	29	72.3	4.9	112.4	6.0	4
	E3 ubiquitin-protein ligase LRSAM1		83.9	5.8	50.3	5.4	3
20	Serum albumin	26	68.5	5.9	86.9	4.0	2
	Titin		3813.7	6.0	78.0	0.2	7
	Heat shock protein beta-1		22.4	6.0	50.4	5.0	1
21	Heat shock protein beta-1	25	22.4	6.0	89.6	8.5	2
22	Titin	24	3813.7	6.0	95.0	0.3	9
	Heat shock protein beta-1		22.4	6.0	55.3	5.0	1
	Nesprin-1		1010.5	5.4	51.1	0.4	4
23	Nesprin-1	23	1010.5	5.4	50.6	0.4	4
24	Titin	22	3813.7	6.0	54.6	0.2	6
	Dystonin		860.1	5.1	51.6	0.7	3
25	Titin	17	3813.7	6.0	73.3	0.2	6
	Peroxiredoxin-5, mitochondrial		22.2	10.2	50.1	10.7	2
26	Nesprin-1	15	1010.5	5.4	54.9	0.7	5
	Protein bicaudal D homolog 2		93.5	5.2	50.2	3.6	3
27	Canalicular multispecific organic anion transporter 1	12	175.4	9.5	50.2	1.9	3

**Table 2:** Medians with standard deviations (SD) estimated for protein phosphorylation intensity (%) of every fraction (season × type of phosphorylated residue).

Band	Protein	Season	Type of P-Residues	Median	SD
7	75 kDa Endoplasmic reticulum chaperone BiP	s	thr	6.515 a	5.751
		os		0.000 b	3.670
12	50 kDa Protein disulfide-isomerase A3	s	ser	7.125 a	5.509
		os		3.105 b	3.224
17	32 kDa Endoplasmic reticulum chaperone BiP	s	tyr	2.985 a	2.605
		os		0.000 b	2.008
		s	thr	3.220 a	2.431
		os		0.000 b	2.202
23	23 kDa Nesprin-1	s	ser	17.555 a	15.126
		os		2.910 b	5.688
		s	tyr	21.055 a	14.799
		os		8.080 b	12.271
25	17 Peroxiredoxin-5, mitochondrial	s	thr	7.860 a	7.746
		os		0.000 b	6.504
26	15 Protein bicaudal D homolog 2	s	thr	4.175 a	3.882
		os		0.000 b	2.699

Different letters indicate statistically significant differences in phosphorylation intensities between season and out of the season (*p* ≤ 0.05).

**Table 3:** Medians with standard deviations (SD) estimated for protein phosphorylation intensity (%) of every fraction (epididymal region × type of phosphorylated residue).

Band	Protein	Region	Type of P-Residues	Median	SD
10	61 kDa Endoplasmic reticulum chaperone BiP, albumin	c1	tyr	1.755 a	2.689
		c2		1.600 a	4.884
		c3		9.345 b	5.459
12	50 kDa Protein disulfide-isomerase A3	c1	thr	11.700 a	10.589
		c2		11.330 a	10.278
		c3		0.000 b	7.530
27	15 kDa Protein bicaudal D homolog 2	c1	ser	4.375 a	4.345
		c2		3.430 a	5.780
		c3		0.000 b	2.546
		c1	tyr	8.430 a	5.903
		c2		3.215 b	7.186
		c3		0.000 c	6.327

Different letters indicate statistically significant differences in phosphorylation intensities between regions of the epididymis (*p* ≤ 0.05).

## Data Availability

The data presented in this study are available upon request from the corresponding author.
